# Supplementary material for: The Arabidopsis Deubiquitylase OTU5 Suppresses Flowering by Histone Modification-Mediated Activation of the Major Flowering Repressors FLC, MAF4, and MAF5
Source: Int J Mol Sci. 2023 Mar 24;24(7):6176. doi: 10.3390/ijms24076176 (PMC10093928; doi:10.3390/ijms24076176)
Supplement: Supplementary file 1 [file ijms-24-06176-s001.zip › Supplemental Figures.pdf]

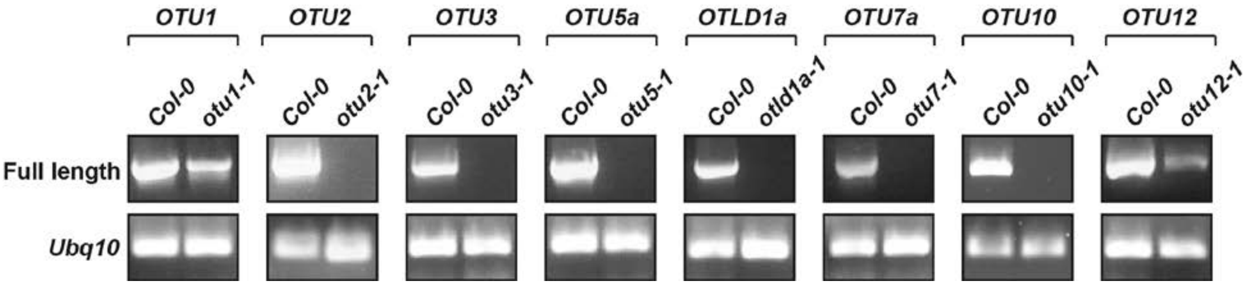

Figure S1. Expression levels for corresponding full-length coding regions for various *OTU* T-DNA insertion mutants. Expression levels of full-length transcripts were examined by RT-PCR in various *OTU* T-DNA insertion mutants and Col-0 (control). The corresponding primer pairs for cloning His-tagged or GST-tagged (for *OTLD1a*) recombinant constructs were used as previous described [9]. The amplification of the *UBQ10*-specific fragment was used as a control with the primer pair UBQ10-5'/UBQ10-3' (Table S4).

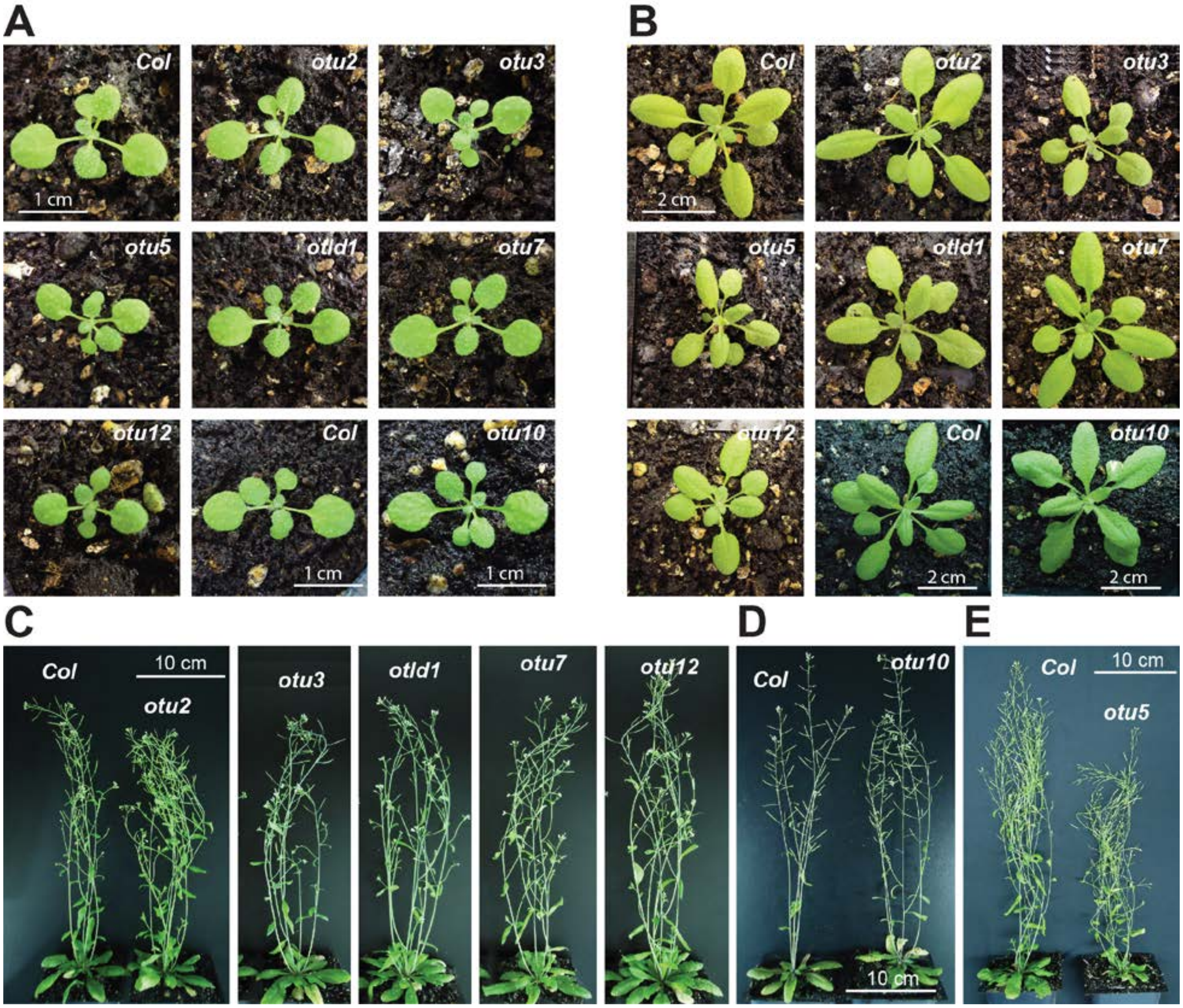

Figure S2. Growth phenotypes at different developmental stages for various *A. thaliana OTU* T-DNA insertion mutants. **(A)** and **(B)** Representative plants at 14 **(A)** and 21 DAS **(B)** for Col-0 and various *OTU* mutants: *otu2-1*, *otu3-1*, *otu5-1*, *otld1-1*, *otu7-1*, *otu10-1*, and *otu12-1*. The *otu10-1* and Col-0 plants were from different growth batches (*bottom*, *middle* and *right panels*). **(C)** to **(E)** Representative plants at 45 DAS for various *OTU* mutants: the *otu2-1*, *otu3-1*, *otld1-1*, *otu7-1*, *otu12-1* **(C)**, *otu10-1* **(D)**, and *otu5-1* **(E)** plants were grown together with Col-0 plants.

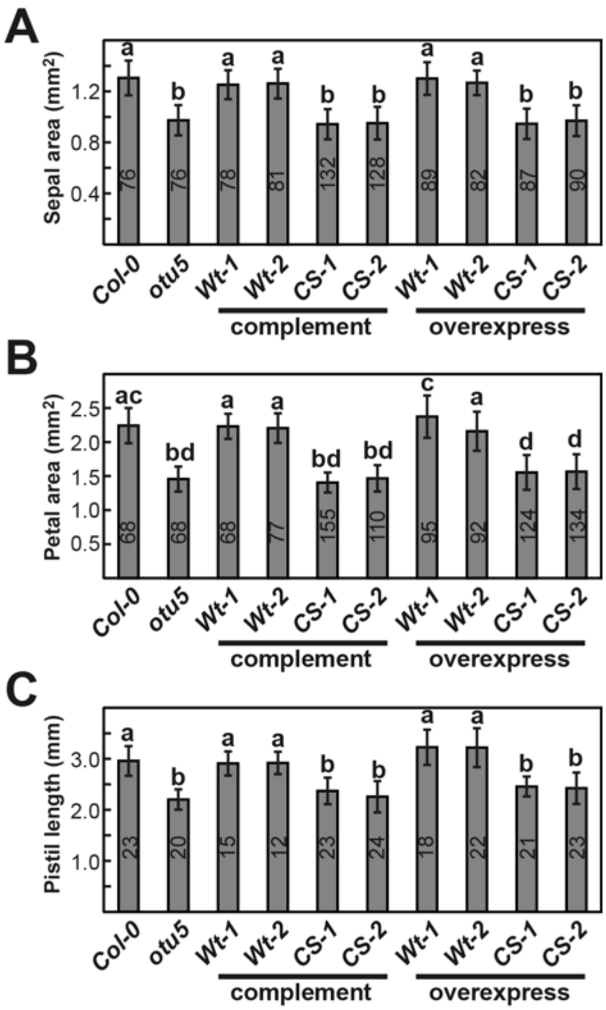

Figure S3. Averaged sepal area, petal area, and pistil length of opened flowers for Col-0, *otu5-1*, and various complemented and overexpressed lines. Averaged sepal area **(A)**, petal area **(B)**, and pistil length **(C)** for various *Arabidopsis* lines. Numbers of sepals, petals, pistils used for average are indicated. Different letters denote significant difference by pairwise comparison using Student's *t* test.

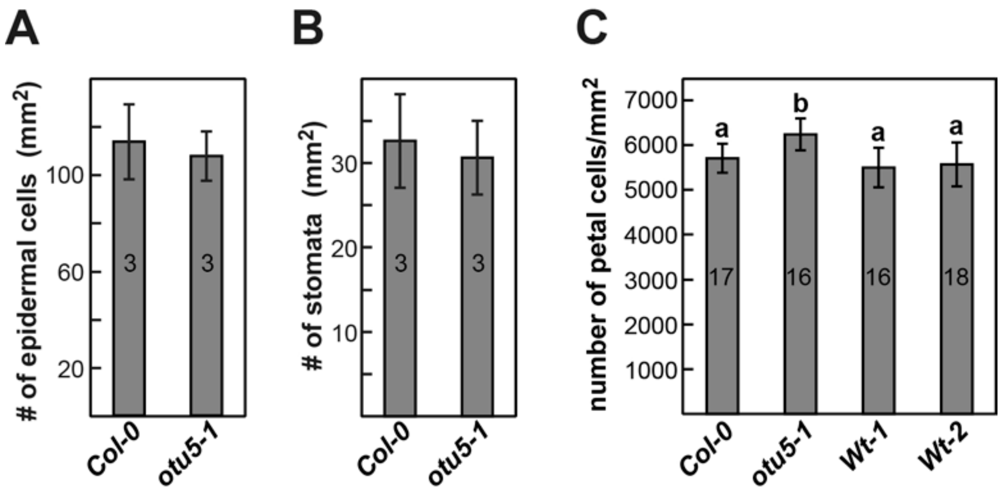

Figure S4. Averaged densities of epidermal cells and stomata for rosette leaves and adaxial epidermal cells of petals in Col-0 and *otu5-1* plants. **(A)** and **(B)** Densities of epidermal cells and stomata were averaged from 32 and 39 unit areas from three 5th rosette leaves for Col-0 and *otu5-1*, respectively. **(C)** Density of adaxial epidermal cells for petals was calculated from 16-18 petals as indicated for Col-0, *otu5-1* and two complemented lines: *Wt-1* and *Wt-2*. Different letters denote significant difference by pairwise comparison using Student's *t* test.

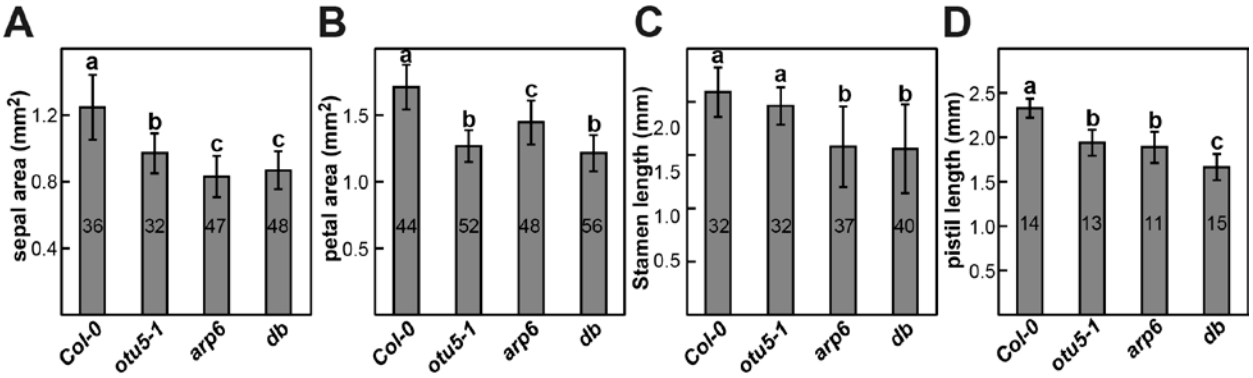

Figure S5. Averaged area or lengths of various floral organs for Col-0, *otu5-1*, *arp6-1*, and the *otu5 arp6* double mutant plants. Averaged sepal area **(A)**, petal area **(B)**, stamen lengths **(C)**, and pistil lengths **(D)** from Col-0, *otu5-1*, *arp6-1*, and *otu5 arp6* (*db*) flowers. Number of various floral organs used were indicated. Different letters denote significant difference by pairwise comparison using Student's *t* test.

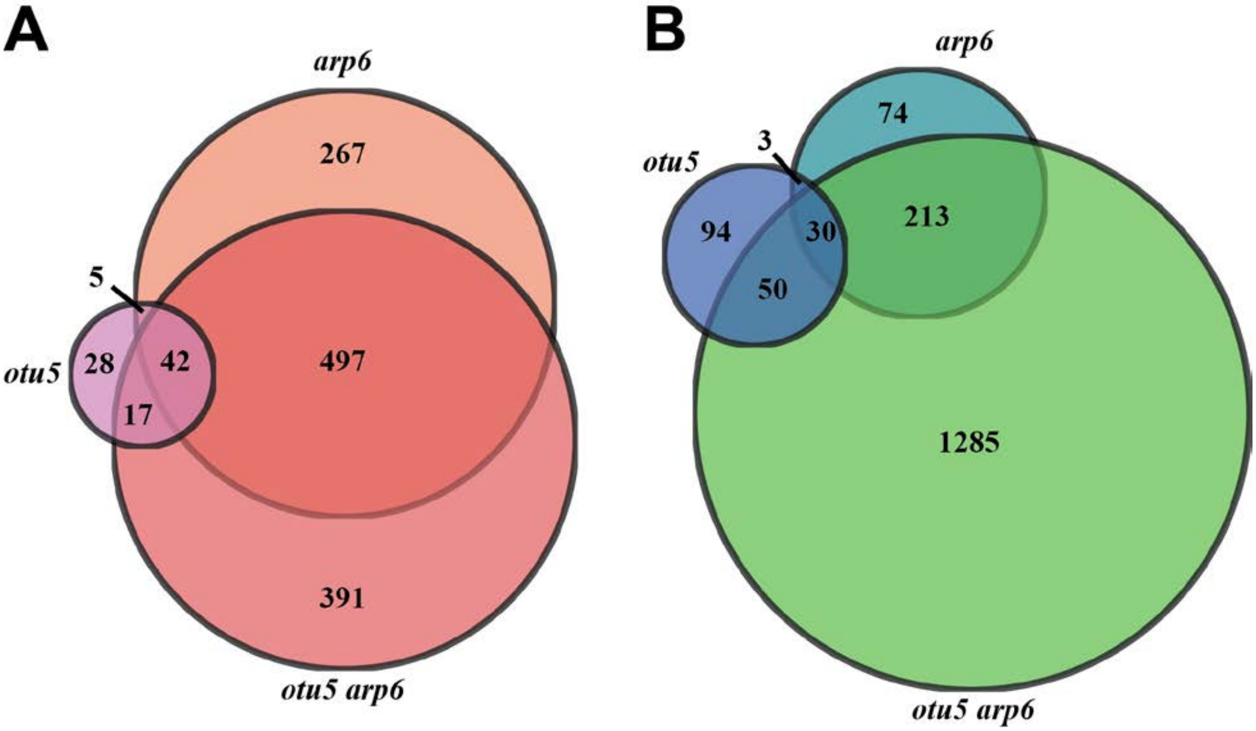

Figure S6. Venn diagrams showing numbers of up- and downregulated DEGs detected from *otu5-1*, *arp6-1* and *otu5 arp6*. **(A)** upregulated and **(B)** downregulated DEG numbers. The numbers not overlapped are unique to specific genotypes and those in overlapped regions are DEG numbers shared between genotypes.
