# Supplementary material for: The Arabidopsis Deubiquitylase OTU5 Suppresses Flowering by Histone Modification-Mediated Activation of the Major Flowering Repressors FLC, MAF4, and MAF5
Source: Int J Mol Sci. 2023 Mar 24;24(7):6176. doi: 10.3390/ijms24076176 (PMC10093928; doi:10.3390/ijms24076176)
Supplement: Supplementary file 1 [file ijms-24-06176-s001.zip › TableS01.pdf]

Table S1. The characterized *A. thaliana* T-DNA insertion lines for genes encoding OTU deubiquitinases.

T-DNA line	Locus	Stock name[a]	Insertion site	RNA/protein expression[b]
otu1-1	At1g28120	Salk_058652	Intron 3, 6 bp upstream of exon 4	KD/KO
otu2-1	At1g50670	Salk_089249	Intron 2, 16 bp upstream of exon 3	KO/KO
otu3-1	At2g38025	Salk_019314	Intron 3, 8 bp downstream exon 3	KO/ND
otu5-1	At3g62940	Salk_032407	Exon 2, 23 bp downstream of *OTU5a* start codon (22 bp upstream of *OTU5b* start codon)	KO/KO
otld1-1	At2g27350	Salk_028707	Exon 8, 34 bp upstream of 3’ exon junction	KO/ND
otu7-1	At5g67170	Salk_032746	Exon 7, 31 bp upstream of intron 7	KO/ND
otu10-1	At5g03330	GABI-Kat 184G03	Intron 4, 1 bp upstream of exon 5	KO/ND
otu12-1	At3g02070	Sslk_129340	Intron 7, 45 bp downstream of exon 7	KD/ND

[a]SALK and GABI-Kat lines were requested from the Arabidopsis Biological Resource Center at Ohio State University (Columbus, OH, USA) and the European Arabidopsis Stock Center at the University of Nottingham (Loughborough, UK), respectively.

[b]KD, knock-down determined by RT-PCR; KO, knock-out determined by RT-PCR or immunoblotting; ND, not determined.
