# Supplementary material for: The Arabidopsis Deubiquitylase OTU5 Suppresses Flowering by Histone Modification-Mediated Activation of the Major Flowering Repressors FLC, MAF4, and MAF5
Source: Int J Mol Sci. 2023 Mar 24;24(7):6176. doi: 10.3390/ijms24076176 (PMC10093928; doi:10.3390/ijms24076176)
Supplement: Supplementary file 1 [file ijms-24-06176-s001.zip › TableS02.pdf]

Table S2. The differentially expressed genes in *otu5-1*.

	Probe	Locus[a]	Fold	Description	GO categories[b]
Downregulated genes					
1	A_84_P11480	AT1G53480	-22.5	MRD1 (*mto1* RESPONDING DOWN 1).	
2	A_84_P24164	AT3G62940	-18.9	OTU5.	
3	A_84_P761415	AT3G42658	-5.9	Member of Sadhu non-coding retrotransposon family. In some natural accessions the allele is methylated and silenced.	
4	A_84_P751526	AT1G72920	-5.6	Putative disease resistance protein (Toll-Interleukin-Resistance-NBS class).	P3/P4
5	A_84_P156615	AT4G12490	-5.2	Bifunctional trypsin-α amylase inhibitor/lipid-transfer protein (LTP)/seed storage 2S albumin superfamily protein.	P2/P4/P10/F2
6	A_84_P785348<br>A_84_P296724	AT5G51190	-4.8<br>-4.2	A member of the ERF (ethylene response factor) subfamily B-3 of ERF/AP2 transcription factor family.	P1/F5/C5
7	A_84_P550153	AT3G30720	-4.3	QUA-QUINE STARCH.	
8	A_84_P545044	AT5G03090	-4.2	Unknown protein.	
9	A_84_P19516	AT4G25470	-3.9	Encodes a member of the DREB subfamily A-1 of ERF/AP2 transcription factor family (CBF2/C-REPEAT/DRE BINDING FACTOR 2).	P5/P9/F5/F8/C5
10	A_84_P17343	AT2G44840	-3.8	A member of the ERF (ETHYLENE RESPONSE FACTOR) subfamily B-3 of ERF/AP2 transcription factor family.	P1/F5/C5
11	A_84_P788437<br>A_84_P171073	AT1G35210<br>AT1G35210	-3.8<br>-3.2	Unknown protein with an unknown function DUF740 (InterPro:IPR008004) domain.	
12	A_84_P784210<br>A_84_P10170<br>A_84_P784401	AT5G10140	-3.5<br>-3.4<br>-3.0	MADS-box protein encoded by FLOWERING LOCUS C - transcription factor that functions as a repressor of floral transition and contributes to temperature compensation of the circadian clock.	P5/P6/P8/F5/F6
13	A_84_P504929<br>A_84_P834641	AT3G29000	-3.5<br>-3.1	Calcium-binding EF-hand family protein.	
14	A_84_P15331	AT2G43620	-3.5	Chitinase family protein.	C3
15	A_84_P103016	AT4G12500	-3.4	Bifunctional inhibitor/lipid-transfer protein/seed storage 2S albumin superfamily protein.	P10/F2
16	A_84_P21874	AT1G27730	-3.3	SALT TOLERANCE ZINC FINGER (STZ), related to Cys2/His2-type zinc-finger proteins found in higher plants.	P1/P3/P5/P7/P15/F5/F6/C5
17	A_84_P13916	AT4G36430	-3.1	Peroxidase superfamily protein.	F1/F12/C2
18	A_84_P21841<br>A_84_P812454	AT1G02930	-3.1<br>-2.5	ATGSTF6, encodes glutathione transferase belonging to the phi class of GSTs.	P4/P7/P15/F4/C2
19	A_84_P20180	AT2G32030	-3.0	GCN5-related Acyl-CoA N-acyltransferases (NAT) superfamily protein.	
20	A_84_P97916	AT4G29780	-2.9	Unknown protein.	
21	A_84_P15931	AT5G47230	-2.9	ERF5, encodes a member of the ERF (ETHYLENE RESPONSE FACTOR) subfamily B-3 of ERF/AP2 transcription factor family.	P1/P5/F5/F8/C5

22	A_84_P809041<br>A_84_P21047	AT2G38530	-2.9<br>-2.6	LTP2, belongs to a family of lipid transfer proteins, involved in lipid transfer between membranes. Similar to other Arabidopsis *LPT* genes but highest similarity to LPT1.	P10/P11/F2
23	A_84_P21109<br>A_84_P807916	AT2G38390	-2.9<br>-2.5	Peroxidase superfamily protein.	P7/F1/F12
24	A_84_P808228<br>A_84_P18366	AT3G20470	-2.7<br>-2.7	GLYCINE-RICH PROTEIN 5 (GRP5), encodes a glycine-rich protein that is expressed more abundantly in immature seed pods than in stems and leaves.	C2
25	A_84_P786490	AT2G46400	-2.7	WRKY DNA-BINDING PROTEIN 46, member of WRKY Transcription Factor; Group III.	P1/F5/C5
26	A_84_P10415	AT1G52030	-2.7	MYROSINASE-BINDING PROTEIN 2 (MBP2), may be involved in metabolizing glucosinolates and forming defense compounds to protect against herbivory. Also similar to lectins and other agglutinating factors. Expressed only in flowers.	P3/P4/P6/F10/C4
27	A_84_P16923	AT5G59820	-2.7	RESPONSIVE TO HIGH LIGHT 41 (RHL41), Encodes a zinc finger protein involved in high light and cold acclimation.	P1/P3/P5/P7/P9/P12/P15/F5
28	A_84_P16942	AT5G64120	-2.6	Encodes a cell wall bound peroxidase that is induced by hypo-osmolarity.	P2/P4/F1/C2/C3
29	A_84_P11274<br>A_84_P825221	AT5G65080	-2.6<br>-2.6	MADS AFFECTING FLOWERING 5 (AGL68/MAF5), upregulated during vernalization and regulates flowering time.	P5/P6/P8/F5/C5
30	A_84_P16848	AT5G39580	-2.6	Peroxidase superfamily protein.	P2/P4/F1/F12
31	A_84_P271180	AT1G53490	-2.5	RING/U-box superfamily protein. Overlapped with AT1G53480.	
32	A_84_P91639	AT2G39030	-2.5	Acyl-CoA N-acyltransferases (NAT) superfamily protein.	
33	A_84_P19239	AT1G44830	-2.5	Encodes a member of the DREB subfamily A-5 of ERF/AP2 transcription factor family. The protein contains one AP2 domain. There are 15 members in this subfamily, including RAP2.1, RAP2.9 and RAP2.10.	F5/C5
34	A_84_P842527	AT5G42800	-2.5	DIHYDROFLAVONOL 4-REDUCTASE, catalyzes the conversion of dihydroquercetin to leucocyanidin in the biosynthesis of anthocyanins.	F9
35	A_84_P199304	AT1G19610	-2.5	LOW-MOLECULAR-WEIGHT CYSTEINE-RICH 78 (LCR78), PDF1.4, encode a PR (pathogenesis-related) protein. Belongs to the plant defensin (PDF) family.	P4
36	A_84_P21958	AT2G43520	-2.5	TRYPSIN INHIBITOR PROTEIN 1 (ATTI1), member of the defensin-like (DEFL) family. Encodes putative trypsin inhibitor protein, which may function in defense against herbivory.	P4/C3
37	A_84_P12264	AT5G58310	-2.4	METHYL ESTERASE 18 (MES18), encodes a protein shown to have methyl IAA esterase activity in vitro.	
38	A_84_P553370	AT1G56240<br>AT1G56242	-2.4	PHLOEM PROTEIN 2-B13 (AtPP2-B13), F-box domain.<br>Potential natural antisense gene, locus overlaps with AT1G56240.	
39	A_84_P18533	AT4G12470	-2.4	AZELAIC ACID INDUCED 1, involved in the priming of salicylic acid induction and systemic immunity triggered by pathogen or azelaic acid. AT4G12490 (#5) and AT4G12500 (#15) also induced.	P2/P4/P10/F2
40	A_84_P602187	AT1G05575	-2.4	Unknown protein, anaerobic respiration.	
41	A_84_P854289<br>A_84_P216738<br>A_84_P805783	AT2G05380	-2.3<br>-2.2<br>-2.0	GLYCINE-RICH PROTEIN 3 SHORT ISOFORM (GRP3S).	
42	A_84_P21275	AT3G50930	-2.3	Cytochrome BC1 synthesis (BCS1), AAA-type ATPase, nucleoside-triphosphatase activity.	

43	A_84_P159845	AT3G28290<br>AT3G28300	-2.3	AT14A, possesses a transmembrane domain and a small region that has sequence similarities to integrins from fungi, insects, and humans. It is localized to plasma membrane and cytoplasm.	C6
44	A_84_P15416	AT2G36970	-2.3	UDP-Glycosyltransferase superfamily protein.	
45	A_84_P813677<br>A_84_P821710	AT5G66390	-2.3<br>-2.0	Peroxidase superfamily protein.	P15/ F1/F12
46	A_84_P22607	AT5G61600	-2.2	ERF104, encodes a member of the ERF (ETHYLENE RESPONSE FACTOR) subfamily B-3 of ERF/AP2 transcription factor family.	P2/P4/F5/F8/C5
47	A_84_P805655<br>A_84_P19508<br>A_84_P805609	AT4G23680<br>AT4G23680<br>AT4G23680	-2.2<br>-2.1<br>-2.1	Polyketide cyclase/dehydrase and lipid transport superfamily protein.	P4
48	A_84_P156125	AT3G61190	-2.2	BON ASSOCIATION PROTEIN 1 (BAP1), encodes a protein with a C2 domain that binds to BON1 in yeast two hybrid analyses. Its ability to bind to phospholipids is enhanced by calcium ions. Involved in maintaining cell homeostasis.	P3/P4/P5/F2
49	A_84_P20704	AT5G59320	-2.2	LIPID TRANSFER PROTEIN 3 (LTP3), encodes a PR (pathogenesis-related) protein. Belongs to the lipid transfer protein (PR-14) family with the following members: At2g38540/LTP1, At2g38530/LTP2, At5g59320/LTP3, At5g59310/LTP4, At3g51600/LTP5, At3g08770/LTP6, At2g15050/LTP7, At2g18370/LTP8, At2g15325/LTP9, At5g01870/LTP10, At4g33355/LTP11, At3g51590/LTP12, At5g44265/LTP13, At5g62065/LTP14, At4g08530/LTP15.	F2/C2/C3
50	A_84_P13815	AT4G19030	-2.2	NOD26-LIKE MAJOR INTRINSIC PROTEIN 1 (NLM1/NIP1;1), an aquaporin whose expression level is reduced by ABA, NaCl, dark, and dessication. It is expressed at relatively low levels under normal conditions. Also functions in arsenite transport and tolerance.	
51	A_84_P810605	AT1G24020	-2.1	MLP-like protein 423 (MLP423), involved in response to biotic stimulus, defense response.	P4
52	A_84_P815273	AT4G23810	-2.1	WRKY53, member of WRKY Transcription Factor; Group III.	P1/P4/F5/F8/C5
53	A_84_P223359	AT1G54000	-2.1	GDSL-like Lipase/Acylhydrolase superfamily protein with lipase activity, hydrolase activity, acting on ester bonds, carboxylesterase activity.	C2
54	A_84_P16072	AT1G65690	-2.1	Late embryogenesis abundant (LEA) hydroxyproline-rich glycoprotein family, NDR1/HIN1-like 25 (HARPIN-INDUCED PROTEIN-RELATED).	
55	A_84_P187524	AT2G47010	-2.1	Unknown protein.	
56	A_84_P161623	AT5G50520<br>AT5G50630	-2.1	Major facilitator superfamily protein.	
57	A_84_P22787	AT1G02920	-2.1	GLUTATHIONE S-TRANSFERASE 7, GLUTATHIONE S-TRANSFERASE 11	P2/P4/P7/F4/C5
58	A_84_P14142	AT5G09480	-2.1	Hydroxyproline-rich glycoprotein family protein.	
59	A_84_P555590	AT4G22212	-2.1	Encodes a defensin-like (DEFL) family protein.	P4
60	A_84_P10964	AT4G11190	-2.1	Disease resistance-responsive (dirigent-like protein) family protein, involved in lignin biosynthetic process, defense response.	P4
61	A_84_P22055	AT2G38380	-2.1	Peroxidase superfamily protein.	P7/F1/F12/C2
62	A_84_P113182	AT4G39670	-2.1	Glycolipid transfer protein (GLTP) family protein.	P10/F2
63	A_84_P21578	AT5G39670	-2.1	Calcium-binding EF-hand family protein.	

64	A_84_P21763	AT1G61580	-2.1	RIBOSOMAL PROTEIN 2 (RPL3B), cytosolic large ribosomal subunit.	
65	A_84_P765129	AT3G56210<br>AT4G04223	-2.0	ARM repeat superfamily protein.<br>Other_RNA	
66	A_84_P15266	AT1G74460	-2.0	GDSL-like Lipase/Acylhydrolase superfamily protein.	
67	A_84_P17864	AT5G58860	-2.0	CYTOCHROME P450, FAMILY 86, SUBFAMILY A, POLYPEPTIDE 1 (CYP86A1).	
68	A_84_P857301<br>A_84_P17256	AT2G40140	-2.0<br>-2.0	SALT-INDUCIBLE ZINC FINGER 2 (SZF2), CZF1.	P1/P2/P4/P5/F5
69	A_84_P826434	AT5G43170	-2.0	ZINC-FINGER PROTEIN 3 (AZF3), The protein is localized to the nucleus and acts as a transcriptional repressor.	P1/P5/P7/F5/F6/C5
70	A_84_P14978	AT5G45380	-2.0	DEGRADATION OF UREA 3 (DUR3) contains InterPro DOMAIN/s: Sodium/solute symporter.	
71	A_84_P789165	AT4G20390	-2.0	Uncharacterized protein family (UPF0497).	
Upregulated genes					
1	A_84_P786320	AT5G35935	9.7	Copia-like retrotransposon family.	
2	A_84_P832727<br>A_84_P829935<br>A_84_P769957	ATMG00370<br>ATCG01000<br>ATCG01130<br>AT2G07739	5.7<br>4.9<br>4.1	Ycf1 protein (InterPro:IPR008896), located in endomembrane system.	
3	A_84_P803861<br>A_84_P12125<br>A_84_P869366	AT5G40340	4.8<br>4.3<br>3.9	Tudor/PWWP/MBT superfamily protein.	C5
4	A_84_P14274	AT1G52690	4.4	Late embryogenesis abundant protein (LEA) family protein.	
5	A_84_P843857<br>A_84_P610444	AT2G22795	4.2<br>3.6	Unknown protein.	
6	A_84_P18172	AT2G19970	3.8	Cysteine-rich secretory proteins, Antigen 5, and Pathogenesis-related 1 protein (CAP) superfamily protein.	C3
7	A_84_P769799	ATCG00800	3.7	chrC:124648-124589, encodes a chloroplast ribosomal protein S3, a constituent of the small subunit of the ribosomal complex	C1
8	A_84_P769282	AT5G40451	3.7	Unknown protein.	
9	A_84_P769991	ATCG00820	3.4	RIBOSOMAL PROTEIN S19, encodes a 6.8-kDa protein of the small ribosomal subunit.	C1
10	A_84_P836393<br>A_84_P16682	AT4G26190	3.2<br>2.3	Haloacid dehalogenase-like hydrolase (HAD) superfamily protein with NLI interacting factor domain (InterPro:IPR004274).	
11	A_84_P12130	AT5G41790	3.1	COP1-INTERACTIVE PROTEIN 1 (CIP1), encodes a protein that physically interacts specifically with the putative coiled-coil region of COP1 in vitro.	P13
12	A_84_P852628	AT5G02600	3.1	SODIUM POTASSIUM ROOT DEFECTIVE 1 (NAKR1), NUCLEAR-ENRICHED PHLOEM COMPANION CELL GENE 6 (NPCC6), heavy metal transport/detoxification superfamily protein.	P6/C5
13	A_84_P243495	AT4G15660	3.0	Thioredoxin superfamily protein, function in electron carrier activity, arsenate reductase (glutaredoxin) activity, protein disulfide oxidoreductase activity, involved in cell redox homeostasis; located in endomembrane system.	F7

14	A_84_P810916<br>A_84_P286290<br>A_84_P856546<br>A_84_P810931	AT5G55660	3.0<br>2.4<br>2.3<br>2.2	DEK domain-containing chromatin-associated protein, GTP binding/RNA binding.	
15	A_84_P853737<br>A_84_P19522	AT4G26630<br>AT4G26630	3.0<br>2.2	DEK domain-containing chromatin-associated protein, GTP binding/RNA binding.	
16	A_84_P20679	AT5G52280	2.9	Myosin heavy chain-related protein, protein transport protein-related.	
17	A_84_P16885	AT5G49360	2.9	BXL1 (BETA-XYLOSIDASE 1); hydrolase, hydrolyzing O-glycosyl compounds, encodes a bifunctional β-D-xylosidase/α-L-arabinofuranosidase required for pectic arabinan modification.	C2/C3
18	A_84_P12986	AT5G04020	2.8	Calmodulin-binding protein-related (PICBP).	
19	A_84_P18215	AT2G32290	2.7	BAM6/BMY5 (BETA-AMYLASE 6).	
20	A_84_P513948	AT4G27595	2.7	Unknown protein with Protein of unknown function domain DUF827 (InterPro:IPR008545).	
21	A_84_P823879<br>A_84_P16877	AT5G47240<br>AT5G47240	2.6<br>2.4	ATNUDT8 (NUDIX HYDROLASE HOMOLOG 8), hydrolase.	P3
22	A_84_P506441	AT1G75310	2.6	AUL1 (AUXILIN-LIKE 1 PROTEIN), heat shock protein binding, with heat shock protein DnaJ, N-terminal domain (InterPro:IPR001623).	
23	A_84_P833235<br>A_84_P832416<br>A_84_P16962	AT5G16730	2.6<br>2.5<br>2.4	Similar to unknown protein (AT3G02930.1), with protein of unknown function domain DUF827 (InterPro:IPR008545), and spectrin/alpha-actinin domain (InterPro:IPR018159).	
24	A_84_P832185<br>A_84_P566564	AT1G13220<br>AT1G13220	2.6<br>2.5	LITTLE NUCLEI2, encodes a nuclear coiled-coil protein related to the carrot peripheral nuclear protein NMCP1 that is involved in the determination of plant nuclear structure.	P14/C5
25	A_84_P853870<br>A_84_P830874<br>A_84_P565111	AT3G05900<br>AT3G05905	2.6<br>2.5<br>2.4	Neurofilament protein-related.<br>Other RNA, potential natural antisense gene, locus overlaps with AT3G05900.	
26	A_84_P836309<br>A_84_P10237	AT5G41140	2.5<br>2.4	Myosin heavy chain-related protein, contains domain PTHR23160 (PTHR23160).	
27	A_84_P192434	AT4G36850	2.5	PQ-loop repeat family protein/transmembrane family protein, contains cystinosin/ERS1p repeat (InterPro:IPR006603).	
28	A_84_P830699	AT1G19835	2.5	Similar to unknown protein *A. thaliana* AT1G47900.1, *Vitis vinifera* GB:CAO48232.1, and Oryza sativa GB:EAY89239.1, with unknown function domain DUF869 (InterPro:IPR008587).	
29	A_84_P560489	AT1G63300	2.4	Myosin heavy chain-related protein, similar to unknown protein *A. thaliana* AT5G41140.1, *V. vinifera* GB:CAO17243.1), contains domain PTHR23160 (PTHR23160), Myosin heavy chain-related protein.	
30	A_84_P825811<br>A_84_P23929	AT2G18700<br>AT2G18700	2.4<br>2.4	TREHALOSE-6-PHOSPHATE SYNTHASE 11 (ATTPS11), encodes an enzyme putatively involved in trehalose biosynthesis. The protein has a trehalose synthase (TPS)-like domain that may or may not be active as well as a trehalose phosphatase (TPP)-like domain.	
31	A_84_P766211<br>A_84_P841901	AT5G35490<br>AT5G35490	2.4<br>2.4	Encodes MRU1 (*mto 1* RESPONDING UP). Up-regulated in *mto1-1* mutant that over-accumulates soluble methionine. A maternally expressed imprinted gene.	
32	A_84_P13690	AT3G46370	2.4	Leucine-rich repeat protein kinase family protein, protein serine/threonine kinase activity, kinase activity, ATP binding; involved in transmembrane receptor protein tyrosine kinase signaling pathway.	
33	A_84_P14748<br>A_84_P785475	AT1G17420	2.4<br>2.2	LOX3 (LIPOXYGENASE 3), encode a lipoxygenase. lipoxygenases (LOXs) catalyze the oxygenation of fatty acids. Iron ion binding /lipoxygenase/metal ion binding /oxidoreductase, acting on single donors with incorporation of molecular oxygen, incorporation of two atoms of oxygen.	P3/P4/ P6

34	A_84_P262140<br>A_84_P834716	AT3G19370	2.3<br>2.1	Plant protein of unknown function (DUF869).	
35	A_84_P831629	AT1G67230	2.3	LITTLE NUCLEI1 (LINC1), encodes a nuclear coiled-coil protein related to the carrot peripheral nuclear protein NMCP1 that is involved in the determination of plant nuclear structure.	P14/C5
36	A_84_P76024<br>A_84_P800042	AT1G17360<br>AT1G17360	2.3<br>2.2	COP1-interacting protein-related (AT1G72410.1).	
37	A_84_P752982	AT1G05320	2.3	Unknown protein with Prefoldin domain (InterPro:IPR009053), best match AT2G32240.1.	
38	A_84_P827080	AT4G04330	2.3	Chaperonin-like RbcX protein, contains chaperonin-like RbcX domain (InterPro:IPR003435).	
39	A_84_P188764	AT4G04320<br>AT4G04330	2.2	Malonyl-CoA decarboxylase family protein.<br>Chaperonin-like RbcX protein, contains chaperonin-like RbcX domain (InterPro:IPR003435).	F11
40	A_84_P132345<br>A_84_P819992	AT4G32340	2.3<br>2.1	Tetratricopeptide repeat (TPR)-like superfamily protein.	
41	A_84_P531612	AT4G04745	2.3	Unknown protein, best match AT4G21902.1.	
42	A_84_P18110	AT1G68790	2.2	LITTLE NUCLEI3 (LINC3).	C5
43	A_84_P19580	AT4G14760	2.2	Kinase interacting (KIP1-like) family protein, contains prefoldin (InterPro:IPR009053) and KIP1-like (InterPro:IPR011684) domains.	C5
44	A_84_P15826	AT5G05600	2.2	2-oxoglutarate (2OG) and Fe(II)-dependent oxygenase superfamily protein.	
45	A_84_P843872	AT1G53430	2.2	Leucine-rich repeat transmembrane protein kinase.	
46	A_84_P22009	AT2G45660	2.2	AGAMOUS-LIKE 20, SUPPRESSOR OF OVEREXPRESSION OF CO 1 (SOC1), controls flowering and is required for CO to promote flowering. It acts downstream of FT. Overexpression of SOC1/AGL20 suppresses not only the late flowering of plants that have functional *FRI* and *FLC* alleles but also the delayed phase transitions during the vegetative stages of development. AGL20/SOC1 acts with AGL24 to promote flowering and inflorescence meristem identity. AGL20 upregulates expression of AGL24 in response to GA.	P5/P6/F5/C5
47	A_84_P521496	AT1G65010	2.2	Unknown protein, putative role in flower development.	P6
48	A_84_P13560	AT2G32150	2.2	Haloacid dehalogenase-like hydrolase (HAD) superfamily protein.	
49	A_84_P503380	AT5G08600	2.2	U3 ribonucleoprotein (UTP) family protein, involved in rRNA processing, located in small-subunit processome.	
50	A_84_P831110<br>A_84_P15196	AT1G03080	2.1<br>2.1	Kinase interacting (KIP1-like) family protein, contains prefoldin (InterPro:IPR009053) and KIP1-like (InterPro:IPR011684) domains.	
51	A_84_P290124	AT2G22860	2.1	PHYTOSULFOKINE 2 PRECURSOR (PSK2).	C3
52	A_84_P230589	AT5G41020	2.1	Myb family transcription factor, contains SANT, DNA-binding (InterPro:IPR001005), homeodomain-like (InterPro:IPR009057), Myb, DNA-binding (InterPro:IPR014778), Myb-like (InterPro:IPR017877), Myb transcription factor (InterPro:IPR015495) domains.	F5
53	A_84_P23923	AT2G37900	2.1	Major facilitator superfamily protein, oligopeptide transport, contains PTR2 family proton/oligopeptide symporter, conserved site (InterPro:IPR018456), oligopeptide transporter (InterPro:IPR000109), major facilitator superfamily, general substrate transporter (InterPro:IPR016196) domains.	

54	A_84_P18414	AT1G02610	2.1	RING/FYVE/PHD zinc finger superfamily protein, with protein of unknown function DUF3675 (InterPro:IPR022143), C3HC4 RING-type zinc finger (InterPro:IPR018957), RING-CH-type (InterPro:IPR011016) domain.	
55	A_84_P149018	AT4G39250	2.1	RADIALIS-LIKE SANT/MYB 2 (RL1, RSM2), contains molecular chaperone, heat shock protein, Hsp40, DnaJ (InterPro:IPR015609), SANT, DNA-binding (InterPro:IPR001005), Myb, DNA-binding (InterPro:IPR014778), homeodomain-like (InterPro:IPR009057), SANT (InterPro:IPR017884) domains.	F5
56	A_84_P22385	AT4G32350	2.1	Regulator of Vps4 activity in the MVB pathway protein, with protein of unknown function DUF292 domain (InterPro:IPR005061).	
57	A_84_P158525	AT1G15940	2.1	Tudor/PWWP/MBT superfamily protein, contains armadillo-type fold (InterPro:IPR016024).	
58	A_84_P846552<br>A_84_P19720	AT5G48600	2.1<br>2.1	STRUCTURAL MAINTENANCE OF CHROMOSOME 3 (SMC3).	C5
59	A_84_P127561	AT4G15680	2.0	Thioredoxin superfamily protein.	F7
60	A_84_P15224	AT1G16130	2.0	WALL ASSOCIATED KINASE-LIKE 2 (WAKL2).	
61	A_84_P15720	AT4G22620	2.0	SAUR-like auxin-responsive protein family.	
62	A_84_P16450	AT3G16000	2.0	MAR BINDING FILAMENT-LIKE PROTEIN 1 (MFP1/MSL1.8).	
63	A_84_P19152	AT2G22610	2.0	Di-glucose binding protein, contains kinesin motor (InterPro:IPR019821), malectin/receptor-like protein kinase (InterPro:IPR021720), kinesin (InterPro:IPR001752) domains.	
64	A_84_P282520	AT3G19050	2.0	PHRAGMOPLAST ORIENTING KINESIN 2 (POK2), one of the two *A. thaliana* homologs isolated in yeast two-hybrid screen for interaction partners of maize gene *TANGLED1* (*TAN1*). Based on sequence homology in their motor domains, POK1 and POK2 belong to the kinesin-12 class, which also includes the well-characterized group of phragmoplast-associated kinesins AtPAKRPs. Both kinesins are composed of an N-terminal motor domain throughout the entire C terminus and putative cargo binding tail domains. The expression domains for POK2 constructs were broader than those for POK1; both are expressed in tissues enriched for dividing cells. The phenotype of *pok1*/*pok2* double mutants strongly resembles that of maize *tan1* mutants, as characterized by misoriented mitotic cytoskeletal arrays and misplaced cell walls.	
65	A_84_P12029	AT4G37220	2.0	Cold acclimation protein WCOR413 family, contain cold acclimation WCOR413 domain (InterPro:IPR008892).	
66	A_84_P855355	AT5G49740<br>AT5G49730	2.0	FERRIC REDUCTION OXIDASE 7 (FRO7), which encodes a chloroplast ferric chelate reductase.<br>FERRIC REDUCTION OXIDASE 6 (FRO6), which encodes a plasma membrane-located ferric chelate reductase.	F3
67	A_84_P529724	AT3G20340	2.0	Unknown protein, expression of the gene is downregulated in the presence of paraquat, an inducer of photoxidative stress.	P15

[a]The yellow- and pink-color highlighted loci are those associated with the enriched Gene Ontology (GO) terms (p < 10E-03), in which the pink-color highlighted are those involved in flower development.

[b]The GO terms are categorized as related to specific biological processes (P1-P15), biochemical activities/molecular functions (F1-F12), and cellular and complex components (C1-C6); see Table S3 for the specific term names.
