# Supplementary material for: The Arabidopsis Deubiquitylase OTU5 Suppresses Flowering by Histone Modification-Mediated Activation of the Major Flowering Repressors FLC, MAF4, and MAF5
Source: Int J Mol Sci. 2023 Mar 24;24(7):6176. doi: 10.3390/ijms24076176 (PMC10093928; doi:10.3390/ijms24076176)
Supplement: Supplementary file 1 [file ijms-24-06176-s001.zip › TableS03.pdf]

Table S3. The enriched GO terms for differentially expressed genes in *otu5-1*.

Cat[a]	Term Name	GOID	Depth	Reference[b]	Count (gene list)[c]	Fsh[d]	*elim*[e]
Specific biological processes							
P1	Response to chitin	0010200	6	127	9 (At1G27730/At2G40140/At2G44840/ At2G46400/At4G23810/At5G43170/At5G47230/ At5G51190/At5G59820)	5.24E-09	5.24E-09
P2	Defense response to fungus	0050832	5	132	7 (At1G02920/At2G40140/At4G12470/At4G12490/ At5G39580/At5G61600/At5G64120)	1.98E-06	1.98E-06
P3	Response to wounding	0009611	4	153	7 (At1G17420/At1G27730/At1G52030/At1G72920/ At3G61190/At5G47240/At5G59820)	5.28E-06	5.28E-06
P4	Defense response	0006952	4	796	19 (At1G02920/At1G02930/At1G17420/ At1G19610/At1G24020/ At1G52030/At1G72920/At2G40140/At2G43520/At3 G61190/At4G11190/At4G12470/At4G12490/At4G22 212/At4G23680/At4G23810/At5G39580/At5G61600 /At5G64120)	2.28E-09	3.81E-05
P5	Response to cold	0009409	4	279	10 (At1G27730/At2G40140/At2G45660/At3G61190/At 4G25470/At5G10140/At5G43170/At5G47230/At5G 59820/At5G65080)	4.63E-07	6.97E-04
P6	Flower development	0009908	4	338	7 (At1g17420/At1G52030/At1g65010/ At2G45660/At5G02600/At5g10140/ At5G65080)	7.50E-04	7.50E-04

P7	Response to salt stress	0009651	5	416	7 (At1G02920/AT1G02930/At1G27730/At2G38380/At2G38390/At5G43170/At5G59820)	0.002451	0.002451
P8	Vernalization response	0010048	5	17	2 (At5G10140/At5G65080)	0.002476	0.002476
P9	Cold acclimation	0009631	5	21	2 (At4G25470/At5G59820)	0.00378	0.00378
P10	Lipid transport	0006869	5	147	5 (At2G38530/At4G12470/At4G12490/AT4G12500/At4G39670)	4.86E-04	0.003951
P11	Phospholipid transfer to membrane	0006649	4	1	1 (At2G38530)	0.004375	0.004375
P12	Photosynthetic acclimation	0009643	7	1	1 (At5G59820)	0.004375	0.004375
P13	Regulation of protein import into nucleus	0042306	5	1	1 (At5G41790)	0.004375	0.004375
P14	Nucleus organization	0006997	6	28	2 (At1G13220/At1G67230)	0.006669	0.006669
P15	Response to oxidative stress	0006979	4	278	5 (At1G02930/At1G27730/At3G20340/At5G59820/At5G66390)	0.007705	0.007705
Specific biochemical activities/molecular functions							
F1	Peroxidase activity	0004601	3	103	6 (At2G38380/At2G38390/At4G36430/At5G39580/At5G64120/At5G66390)	6.38E-06	6.38E-06
F2	Lipid binding	0008289	3	229	7 (At2G38530/At3G61190/At4G12470/At4G12490/At4G12500/At4G39670/At5G59320)	7.07E-05	7.07E-05
F3	Ferric-chelate reductase activity	0000293	6	8	2 (At5G49730/At5G49740)	5.23E-04	5.23E-04
F4	Glutathione binding	0043295	4	10	2 (At1G02920/At1G02930)	8.36E-04	8.36E-04
F5	Sequence-specific DNA binding	0003700	3	1681	17	0.001127	0.001127

	transcription factor activity				(At1G27730/At1G44830/At2G40140/At2G44840/At2G45660/At2G46400/At4G23810/At4G25470/At4G39250/At5G10140/At5G41020/At5G43170/At5G47230/At5G51190/At5G59820/At5G61600/At5G65080)		
F6	Transcription repressor activity	0016564	3	51	3 (At1G27730/At5G10140/At5G43170)	0.001465	0.001465
F7	Arsenate reductase (glutaredoxin) activity	0008794	5	14	2 (At4G15660/At4G15680)	0.001671	0.001671
F8	Transcription activator activity	0016563	3	129	4 (At4G23810/At4G25470/At5G47230/ At5G61600)	0.002535	0.002535
F9	Dihydrokaempferol 4-reductase activity	0045552	6	1	1 (At5G42800)	0.004375	0.004375
F10	Thioglucosidase binding	0010180	5	1	1 (At1G52030)	0.004375	0.004375
F11	Malonyl-CoA decarboxylase activity	0050080	6	1	1 (At4G04320)	0.004375	0.004375
F12	Heme binding	0020037	4	290	5 (At2G38380/At2G38390/At4G36430/At5G39580/At5G66390)	0.009147	0.009147
**Specific cellular/complex components**							
C1	Plastid small ribosomal subunit	0000312	5	19	2 (AtCG00800/AtCG00820)	0.003096	0.003096
C2	Cell wall	0005618	4	554	8 (At1G02930/At1G54000/At2G38380/At3G20470/At4G36430/At5G49360/At5G59320/At5G64120)	0.003139	0.003139
C3	Extracellular region	0005576	2	439	7 (At2G19970/At2G22860/At2G43520/At2G43620/At5G49360/At5G59320/At5G64120)	0.003298	0.003298
C4	Thioglucosidase complex	0010169	4	1	1 (At1G52030)	0.004375	0.004375
C5	Nucleus	0005634	5	2435	20 (At1G02920/At1G13220/At1G27730/At1G44830/At1G67230/At1G68790/At2G44840/At2G45660/At2G	0.004791	0.004791

					46400/At4G14760/At4G23810/At4G25470/At5G02600/At5G40340/At5G43170/At5G47230/At5G48600/At5G51190/At5G61600/At5G65080)		
C6	Integrin complex	0008305	5	2	1 (At3G28300)	0.00873	0.00873

[a]Cat, GO terms are classified as being involved in specific biological processes (P1-P15), specific biochemical activities/molecular functions (F1-F12), and specific cellular and complex compartments (C1-C6) based on the TAIR GO annotations.

[b]The reference referred to the number of loci annotated in the TAIR10 genome release with the specific GO term.

[c]Loci indicated in green and red colors are down- and up-regulated, respectively.

[d]Fsh, Fisher's exact test.

[e]*elim*, TopGO *elim* algorithm.
