# Supplementary material for: The Arabidopsis Deubiquitylase OTU5 Suppresses Flowering by Histone Modification-Mediated Activation of the Major Flowering Repressors FLC, MAF4, and MAF5
Source: Int J Mol Sci. 2023 Mar 24;24(7):6176. doi: 10.3390/ijms24076176 (PMC10093928; doi:10.3390/ijms24076176)
Supplement: Supplementary file 1 [file ijms-24-06176-s001.zip › TableS04.pdf]

**Table S4.** PCR primer pairs used in this study.	
**Gene/stock /construct/PCR fragment**	Primer pairs and note
**Amplification of T-DNA insertion junctions and corresponding wild-type genes**	
otu1-1	Wild-type<br>T_OTU1_5’: GAACCTCTGTCTAGTTTAGCTGCAGAG<br>T_OTU1_3’: AGAATGTCATCTAGTTGCTCAAGGAAC<br>T-DNA junction<br>pROK2_LBa1: TGATGGTTCACGTAGTGGGCCATCGCCCTG<br>T_OTU1_3’: AGAATGTCATCTAGTTGCTCAAGGAAC
otu2-1	Wild-type<br>T-OTU2-5’: CTTTCATCGCGTTTGGTGTTCATCTTC<br>T-OTU2-3’: GTTGCTTGCAACTGCTGCTGCTATCAC<br>T-DNA junction<br>T-OTU2-5’: CTTTCATCGCGTTTGGTGTTCATCTTC<br>pROK2_LBa1: TGATGGTTCACGTAGTGGGCCATCGCCCTG
otu3-1	Wild-type<br>T-OTU3_03: GGCTTCGCTAGATTCGAGCTTGTTTC<br>T-OTU3_06: GTACTTCTCCCTTTCCTTGGGGTCATTG<br>T-DNA junction<br>T-OTU3_03: GGCTTCGCTAGATTCGAGCTTGTTTC<br>pROK2_LBa1: TGATGGTTCACGTAGTGGGCCATCGCCCTG
otu5-1	Wild-type<br>T_OTU5_5’: GGGGGTTTGGTCTTGTTTCTAGAGTTG<br>T_OTU5_3’: CCAGCTATAGCTCTAACTAGAGTGTCG<br>T-DNA junction<br>pROK2_LBa1: TGATGGTTCACGTAGTGGGCCATCGCCCTG<br>T_OTU5_3’: CCAGCTATAGCTCTAACTAGAGTGTCG
otld1-1	Wild-type<br>T_OTU6_5endA: GGACTTGGATTTAGTAGCCTTAGTGGG<br>T_OTU6_3endA: TGCTGGTTTCGAATCGCTGCCTGAAGG<br>T-DNA junction<br>T_OTU6_5endA: GGACTTGGATTTAGTAGCCTTAGTGGG<br>pROK2_LBa1: TGATGGTTCACGTAGTGGGCCATCGCCCTG
otu7-1	Wild-type<br>T-OTU7_03: AACACTACAACAGTGTGCGTTCCAAGG<br>T-OTU7_04: TATCATCGGTCTGTGTAGTGCACTTGG<br>T-DNA junction<br>T-OTU7_03: AACACTACAACAGTGTGCGTTCCAAGG<br>pROK2_LBa1: TGATGGTTCACGTAGTGGGCCATCGCCCTG
otu10-1	Wild-type<br>184G03_5' : GGAGAAATACCTCCGGAAGAAGAAG<br>184G03_3' : ATGGAAGCTAGCAAAGATTCTCAA

	T-DNA junction<br>184G03_3' : ATGGAAGCTAGCAAAGATTCTCAA<br>GABI_LB: CCCATTTGGACGTGAATGTAGACAC,<br>GABI_LB4: CACGGATGATCTCGCGGAGGGTAG
otu12-1	Wild-type<br>T_OTU12_5endB: GAATGGGGAGACCATATTACCCTACAAG<br>T_OTU12_3endB: CTCTTTGGCTTATGCTGAACTGGAGCAG<br>T-DNA junction<br>pROK2_LBa1: TGATGGTTCACGTAGTGGGCCATCGCCCTG<br>T_OTU12_3endB: CTCTTTGGCTTATGCTGAACTGGAGCAG
fld-6	Wild-type<br>fld6-F: TCTCCCTTGGGGCAGCACTCGAGAC<br>fld6-R: CATCGCCTGAAGCTCCCACTGCAAC<br>T-DNA junction:<br>fld6-F: TCTCCCTTGGGGCAGCACTCGAGAC<br>LB3: TAGCATCTGAATTTCATAACCAATCTCGATACAC
arp6-1	Wild-type<br>ARP6-1-S: GTTCTTCCTGATGGTGTTACACATA<br>ARP6-1-A: GGCATGAGTTTATAGCTCGGACAAT<br>T-DNA junction:<br>LB3: TAGCATCTGAATTTCATAACCAATCTCGATACAC<br>ARP6-1-A: GGCATGAGTTTATAGCTCGGACAAT
hub1-4	Wild-type<br>HUB1-2 RP: AGCTCCGACAAGAACTCAGTG<br>HUB1-2 LP TGGGAAAACATGGTATTGAGG<br>T-DNA junction:<br>HUB1-2 RP: AGCTCCGACAAGAACTCAGTG<br>pROK2_LBa1: TGATGGTTCACGTAGTGGGCCATCGCCCTG
hub1-5	Wild-type<br>HUB1-1 LP: TTTTCTGTTTCAGGGATGTCG<br>RP-1: GAGAGACCGAAGACGAGAAACTTTC<br>T-DNA junction:<br>pROK2_LBa1: TGATGGTTCACGTAGTGGGCCATCGCCCTG<br>RP-1: GAGAGACCGAAGACGAGAAACTTTC
**RT-PCR**	
FVE	FVE01: AAGATTCTCAGACGCCGTCGTCTC<br>FVE02: ACGGTTTGGTTGGGTTTCAACATC
FLD	FLD01: GAGCACAGATCTCGATACCTTTGG<br>FLD02: TCGTAATCATCGCCTGAAGCTCCC
FPA	FPA01: TGTTAGCGAGCCTCTCAGAATACC<br>FPA02: GAAACAGGATGATTTGACGAACCC
FCA	FCA01: CCCGTTAGGTGGTTATGGTGTTCC<br>FCA02: AGCGGTTGCTGCTGGGAACGAGGG

LD	LD01: TCTTGTTTATGCACTAACTTCGGG<br>LD02: CTGGTTCCCTAACTGCATACGAG
FY	FY01: AGCAGCAAGGGTATCAGCAACAAC<br>FY02: CATCTGTGGACGGTTGAAACCACC
FLC	FLC-01: GCTTCTCCTCCGGCGATAACCTGG<br>FLC-02: TGTCGGAGATTTGTCCAGCAGGTG
MAF1	MAF1-01: TGATGATGGAGTATATCGAGTCCC<br>MAF1-02: AATCCAATCCGTACATTCAGACAC
MAF2	MAF2-01: TAATGATGGGGGAAGTGAAGTCCC<br>MAF2-02: CTGTAAGTTTAAGGTGAAAGCTCAGCC
MAF3	MAF3-01: TAATGATGGAGGATATGAAGTCAC<br>MAF3-02: GAACTCTGATATTTGTCTACTAAGGTAC
MAF4	MAF4-01: GCTGCTGAGAGAGGAGAACCAGAG<br>MAF4-02: TGGATGACTTTTCCGTAGCAGGGGGAAG
MAF5	MAF5-01: AGTTGCTGAAAGAAAAGAACAAGGT<br>MAF5-02: TCCTGTCTTCCAAGGTAACACAAAGG
CO	CO01: GAGTATGGACTACAAATTCACAGG<br>CO02: GGTCAGGTTGTTGCTCTACTGTCC
SOC1	SOC1-01: CATAGATCGTTATCTGAGGCATAC<br>SOC1-02: CTTTCTTGAAGAACAAGGTAACCC
FT	FT-01: AGACGTTCTTGATCCGTTTAATAGATC<br>FT-02: CACTGTTTGCCTGCCAAGCTGTCG
UBQ10	UBQ10-5’: GTGGTGGTTTCTAAATCTCGTCTCTG<br>UBQ10-3’: GAAGAAGTTCGACTTGTCATTAGAAAG
**Verification of flowering mutants**	
FRIGIDA	FRI-SF2_f: AGATTTGCTGGATTTGATAAGG<br>FRI-SF2_r: GAAATTCACCGAGTGAGAACAGA<br>(Wild-type and mutant *FRI* were distinguished by the size differences of *Rsa*I-digested fragment)
ld-1	ld1-F: ACCTCTCCACAAAGCATCTCCTGA<br>ld1-R: GCAACAGTTTTGGACCCTGTGACGA<br>(primers designed to differentiate a G to T mutation at the 5’ splicing donor site of intron 8 in *ld-1*)
fve-4	FVE4-F: ACCGACAGTCCTGATGTGAGTGCTG<br>FVE4-R: GGTTCCGTTGGGCACATGGCAAGAG<br>(primers designed to differentiate a G to A mutation at 11th residue of exon 6 in *fve-4*)
**Complementation, overexpression and transient expression constructs including intermediates**	
**pET28a-OTU5a-C192S**	OTU5a-C192S-T: GATTAAACCAGACGGACATAGCCTTTACCGTGCTGTTG<br>OTU5a-C192S-B: CAACAGCACGGTAAAGGCTATGTCCGTCTGGTTTAATC<br>(site-mutagenized from pET28a-OTU5a)
**pENTR221-OTU5a_C**	OTU5a_CF:<br>GGGG**ACAAGTTTGTACAAAAAAGCAGGCT**TCATGGCAGACAATAAAACATCTGAAG<br>OTU5a_CR:<br>GGGG**ACCACTTTGTACAAGAAAGCTGGGT**ATCATCCGGTGATGTTGTTGACGAGCAC

	(The bold-face letters are attB1 and attB2 sites and the underlined are 5’ and 3’ OTU5-specific sequence; template pET28a-OTU5a)
**pENTR221-OTU5a-C192S_C**	OTU5a_CF:<br>GGGG**ACAAGTTTGTACAAAAAAGCAGGCT**TCATGGCAGACAATAAAACATCTGAAG<br>OTU5a_CR:<br>GGGG**ACCACTTTGTACAAGAAAGCTGGGT**ATCATCCGGTGATGTTGTTGACGAGCAC<br>(template pET28a-OTU5a-C192S)
**OTU5a-eGFP**	OTU5a-X: TCCCCCGGGATGGCAGACAATAAAACATCTGAAG<br>OTU5-E: CATGAATTCTCCGGTGATGTTGTTGACGAGCAC<br>(Amplified fragment was cloned into p2X35S-TEV-eGFP-35ST to express OTU5a-eGFP, see methods)
**OTU5b-eGFP**	OTU5b-X: TCCCCCGGGATGGATGCTTCTCAGGAGCAAC<br>OTU5-E: CATGAATTCTCCGGTGATGTTGTTGACGAGCAC<br>(Amplified fragment was cloned into p2X35S-TEV-eGFP-35ST to express OTU5b-eGFP, see methods)
**eGFP-OTU5a**	OTU5a-B: TCCGGATCC ATGGCAGACAATAAAACATCTGAAG<br>OTU5-S: CATGTCGACTCATCCGGTGATGTTGTTGACGAGCAC<br>(Amplified fragment was cloned into p2X35S-TEV-eGFP-35ST to express eGFP-OTU5a, see methods)
**eGFP-OTU5b**	OTU5b-B: TCCGGATCCATGGATGCTTCTCAGGAGCAAC<br>OTU5-S: CATGTCGACTCATCCGGTGATGTTGTTGACGAGCAC<br>(Amplified fragment was cloned into p2X35S-TEV-eGFP-35ST to express eGFP-OTU5b, see methods)
**RT-qPCR**	
UBQ10	F: GTGGTGGTTTCTAAATCTCGTCTCTG<br>R: GAAGAAGTTCGACTTGTCATTAGAAAG
FLC	F: CCGAACTCATGTTGAAGCTTGTTGAG<br>R: CGGAGATTTGTCCAGCAGGTG
MAF1	F: ACGTTGCTGGCAACAGATGA<br>R: AATTGAGCAGCGGGAGAGTC
MAF2	F: ATGGCTCCGGCAACAAAGTA<br>R: AAGGTGAAAGCTCAGCCGTT
MAF3	F: AAACACATTTTGGGTCCCCG<br>R: ACCTTTGCGTCGTTTGGAGA
MAF4	F: TTTCGCACAAGGAGTTGCT<br>R: GCTGCTCTTCCAGGGACTTTA
MAF5	F: GAAACAGGGGATGAAAGAGCAGTA<br>R: TGGGCTGTGGCCAGAGCTAT
CO	F: CATTAACCATAACGCATACATTTCATC<br>R: TCCGGCACAACACCAGTT
SOC1	F: ATCGAGTCAGCACCAAACCG<br>R: AGCTGTTGCTCAATCTGTTGC
FT	F: ATCTCCATTGGTTGGTGACTGATA

	R: GCCAAAGGTTGTTCCAGTTGTAG
FVE	F: ATGGCGGATGAGTGACTTGA<br>R: ACTCTTAAGGCTTGGAGGCAC
FLD	F: TTAAAGCCGAGCGAACTGGT<br>R: GCGGCAACGGTCCTTTAATC
FPA	F: AGGCCCCTCAGAGGTACAAA<br>R: CTTGTGCAGGAGATGGGAGG
FCA	F: GCTCGGACACATATACCCGT<br>R: GCTCCTCGTCTCATGTACCTC
LD	F: CGGTTTGCGTATTGGTCTTCG<br>R: ACGACGACATCTTGGAGCTG
FY	F: CGCACTGTCTGCGGGATAAA<br>R: TGCATATTGGAACAGAACAAAAACC
**ChIP-qPCR**	
FLC1	F: ATTTAGCAACGAAAGTGAAAACTAAGG<br>R: GCCACGTGTACCGCATGAC
FLC2	F: AGAAATCAAGCGAATTGAGAACAA<br>R: CGTTGCGACGTTTGGAGAA
FLC3	F: AATTGCATGTCATTCACGATTTG<br>R: TGAAACTTCACTCAACAACATCGA
FLC4	F: TTATCGCCCTTAATCTTATCATCGT<br>R: AAGATTGAGGTTGTGGATTGTCAA
FLC5	F: CCGCCACATCATCATTATCATC<br>R: ACAAGGTTTTTTCCAGCGATAGA
FLC6	F: AAGCCAGCGCTATCACTAAACTTT<br>R: TCGGCAGATTGAAAATGACATT
FLC7	F: CATCTCTCCAGCCTGGTCAAG<br>R: GGCTTTAAGATCATCAGCATGCT
FLC8	F: AGCCAGGTAACGAAAGCTACATTT<br>R: ACATGGACATTGGACACACAACA
FLC9	F: CATCATGTGGGAGCAGAAGCT<br>R: CGGAAGATTGTCGGAGATTTG
FLC10	F: CGTATGGTTGGCATGGGT TT<br>R: ACTAAGCTTATTGGGCCGAATG
MAF4-1	F: AAAAGTACGGATTGGATTCAAAAGA<br>R: GGAAAACCAAAGGAATTAAGAAGCT
MAF4-2	F: GACCAACGCGCCACAAG<br>R: CGGTGCGTTTTTAATAGGAGTTTAG
MAF4-3	F: TGGTGTCCAAAGTCCAGAGATG<br>R: CCTTGCAACAAATGAAAACAATAGA
MAF4-4	F: CCGAATTGAGACCTTGTAGAAGTAGA<br>R: ATCAAGCATTTGTGGTGTTAAGTATGA
MAF4-5	F: GTTGTTTTCCTTTTCTGTTGTTTATCTATG

	R: ATACTTACATTATCGCTTTTCGCTTCT
MAF4-6	F: GCGGAAAGCCGGTAAAAGAC<br>R: CGAATCTGGGCTTAACAGTAACAGT
MAF5-1	F: CTTTTTCTCACTGTTTTGTCTTTATTCAGT<br>R: CCCTGAGGGCGCGTTTAT
MAF5-2	F: TGGTGGCGGAAGATGTACTCT<br>R: TCCCTGCAACAAATTGAAACAA
MAF5-3	F: AATTTGGCTTATTCTGTGAGTGCTT<br>R: TTCAGAAGTTGGTGAAATCATATTGAC
MAF5-4	F: CGGATCTTGCCTGACTTTGG<br>R: GCAACAAGACATTGAGGATTG TG
MAF5-5	F: TTTGCGGCCCATTTAGAGAT<br>R: TCCGAGGAGACAACCAGTGTT
Actin2	F: GATGAGGCAGGTCCAGGAATC<br>R: AACCCCAGCTTTTTAAGCCTTT
