# Supplementary material for: The Arabidopsis Deubiquitylase OTU5 Suppresses Flowering by Histone Modification-Mediated Activation of the Major Flowering Repressors FLC, MAF4, and MAF5
Source: Int J Mol Sci. 2023 Mar 24;24(7):6176. doi: 10.3390/ijms24076176 (PMC10093928; doi:10.3390/ijms24076176)
Supplement: Supplementary file 1 [file ijms-24-06176-s001.zip › TableS06.pdf]

Table S6. Analyses of differential expressed genes of *otu5-1*, *arp6-1*, and *otu5 arp6*.

DEGs[a]		Overlapped with other genotypes[b]			Expression pattern comparison with other genotypes[c]			
					vs arp6		vs otu5	
genotype	up	*otu5-1*	*arp6-1*	*otu5 arp6*	up	Down or unchanged	up	Down or unchanged
otu5-1	92	-	47 (51.1)	59 (64.1)	61 (66.3)	31 (33.7)		
arp6-1	811	47 (5.8)	-	539 (66.5)			162 (20.0)	649 (80.0)
otu5 arp6	947	59 (6.2)	539 (56.9)	-	758 (80.0)	189 (20.0)	247 (26.1)	700 (73.9)
DEGs		Overlapped with other genotypes			Expression pattern comparison with other genotypes			
					vs arp6		vs otu5	
genotype	down	*otu5-1*	*arp6-1*	*otu5 arp6*	down	up or unchanged	down	up or unchanged
otu5-1	177	-	33 (18.6)	80 (45.2)	50 (28.2)	127 (71.8)		
arp6-1	320	33 (10.3)	-	243 (75.9)			82 (25.6)	238 (74.4)
otu5 arp6	1578	80 (5.1)	243 (15.4)	-	442 (28.0)	1136 (72.0)	198 (12.5)	1380 (87.5)

[a]Up- and down-regulated DEGs were calculated on all loci matched with microarray probes, redundant probes were presented by the most up- or down-regulated locus with $p < 0.05$; unmatched, chloroplast, and mitochondria probes were excluded.
[b]Calculated from the Venn diagrams (Figure S6), numbers in parentheses are percentages.
[c]The $\log_2$ of expression fold ≥ 0.2 and ≤ -0.2 are arbitrarily defined as up- and down-regulated and the rest are unchanged.
