# Supplementary material for: The Arabidopsis Deubiquitylase OTU5 Suppresses Flowering by Histone Modification-Mediated Activation of the Major Flowering Repressors FLC, MAF4, and MAF5
Source: Int J Mol Sci. 2023 Mar 24;24(7):6176. doi: 10.3390/ijms24076176 (PMC10093928; doi:10.3390/ijms24076176)
Supplement: Supplementary file 1 [file ijms-24-06176-s001.zip › TableS07.pdf]

Table S7. Comparison of log_2 fold change of 42 upregulated loci that are overlapped between *otu5*, *arp6*, and *otu5 arp6* mutants[a].

Locus	probe ID	*otu5*	*arp6*	*db*	*db - otu5*	*db - arp6*	description
AT1G02660	A_84_P12738	1.341	2.782	2.187	0.846	-0.594	alpha/beta-Hydrolases superfamily protein
AT1G08090	A_84_P18587	1.178	2.267	1.578	0.400	-0.689	nitrate transporter 2:1
AT1G12610	A_84_P16247	2.057	1.947	2.097	0.040	0.151	Integrase-type DNA-binding superfamily protein
AT1G21910	A_84_P20470	1.386	2.338	1.613	0.227	-0.726	Integrase-type DNA-binding superfamily protein
AT1G27045	A_84_P836321	1.112	1.728	1.597	0.485	-0.130	Homeobox-leucine zipper protein family
AT1G50040	A_84_P10518	1.329	2.293	2.534	1.204	0.241	formin-like protein, putative (DUF1005)
AT1G65490	A_84_P57860	1.346	1.761	1.993	0.647	0.232	STMP5, a secreted peptide which functions in plant growth and pathogen defense
AT1G70260	A_84_P839203	1.471	1.852	1.016	-0.455	-0.836	nodulin MtN21 /EamA-like transporter family protein
AT1G76650	A_84_P14331	1.083	2.909	2.793	1.710	-0.115	calmodulin-like 38
AT1G79245	A_84_P798768	1.181	1.189	1.025	-0.156	-0.163	pseudogene of Winged helix-turn-helix transcription repressor DNA-binding protein
AT1G79245	A_84_P803930	1.295	1.340	1.207	-0.088	-0.133	pseudogene of Winged helix-turn-helix transcription repressor DNA-binding protein
AT2G17230	A_84_P67674	3.358	3.551	3.963	0.605	0.412	EXORDIUM like 5
AT2G17230	A_84_P800917	1.279	1.336	1.727	0.448	0.390	EXORDIUM like 5
AT2G17850	A_84_P791417	1.891	1.722	1.348	-0.543	-0.374	Rhodanese/Cell cycle control phosphatase superfamily protein
AT2G28780	A_84_P297814	1.418	1.581	1.297	-0.121	-0.284	P-hydroxybenzoic acid efflux pump subunit
AT2G34600	A_84_P610481	3.268	2.796	2.792	-0.476	-0.004	jasmonate-zim-domain protein 7
AT2G39510	A_84_P20167	2.250	1.046	1.806	-0.445	0.759	nodulin MtN21 /EamA-like transporter family protein
AT2G47520	A_84_P23870	1.112	1.477	1.708	0.596	0.231	ETHYLENE RESPONSE FACTOR 71 (ERF71), a member of the ERF/AP2 subfamily B-2
AT3G19680	A_84_P56760	1.677	2.380	2.560	0.883	0.180	hypothetical protein (DUF1005)
AT3G19680	A_84_P784688	1.753	2.388	2.698	0.945	0.310	hypothetical protein (DUF1005)
AT3G19680	A_84_P799394	1.281	2.110	2.413	1.131	0.303	hypothetical protein (DUF1005)
AT3G50060	A_84_P20325	1.046	1.577	1.595	0.549	0.018	myb domain protein 77
AT3G55980	A_84_P13733	1.178	2.127	1.622	0.444	-0.505	salt-inducible zinc finger 1

AT3G57520	A_84_P806643	1.089	1.030	1.395	0.307	0.365	seed imbibition 2 (SIP2), a raffinose-specific alpha-galactosidase
AT3G59080	A_84_P15635	1.077	2.400	2.137	1.060	-0.262	Eukaryotic aspartyl protease family protein
AT4G01250	A_84_P16606	1.078	1.691	1.055	-0.023	-0.636	WRKY family transcription factor
AT4G08950	A_84_P13790	1.460	2.764	2.937	1.477	0.173	Phosphate-responsive 1 family protein
AT4G08950	A_84_P811890	1.350	2.526	2.081	0.732	-0.445	Phosphate-responsive 1 family protein
AT4G08950	A_84_P811894	1.158	2.621	2.385	1.228	-0.236	Phosphate-responsive 1 family protein
AT4G19520	A_84_P835962	1.092	2.064	1.579	0.488	-0.484	disease resistance protein (TIR-NBS-LRR class) family
AT4G32480	A_84_P59510	1.289	1.670	2.186	0.897	0.516	a putative sugar phosphate exchanger (DUF506)
AT4G32480	A_84_P818265	1.497	1.800	2.291	0.794	0.491	a putative sugar phosphate exchanger (DUF506)
AT4G32800	A_84_P20495	1.056	1.898	1.902	0.847	0.004	Integrase-type DNA-binding superfamily protein
AT4G33070	A_84_P22388	1.209	1.229	1.245	0.036	0.016	Thiamine pyrophosphate dependent pyruvate decarboxylase family protein
AT4G34150	A_84_P853187	1.081	2.309	1.477	0.396	-0.832	Calcium-dependent lipid-binding (CaLB domain) family protein
AT4G34410	A_84_P11046	3.224	3.208	2.716	-0.509	-0.492	redox responsive transcription factor 1
AT4G37260	A_84_P14864	1.021	1.450	1.229	0.208	-0.221	myb domain protein 73
AT4G39250	A_84_P149018	1.646	1.630	2.391	0.744	0.760	RAD-LIKE 1/RADIALIS-LIKE SANT/MYB 2/RSM2
AT5G08150	A_84_P560990	1.835	2.870	2.780	0.945	-0.090	suppressor of phytochrome b 5
AT5G10040	A_84_P157965	1.764	1.935	1.173	-0.590	-0.761	transmembrane protein
AT5G10040	A_84_P787492	1.648	1.887	1.359	-0.289	-0.528	transmembrane protein
AT5G14180	A_84_P11129	1.234	1.207	1.674	0.439	0.466	Myzus persicae-induced lipase 1
AT5G16570	A_84_P52990	1.157	1.172	1.327	0.170	0.155	glutamine synthetase 1;4
AT5G20250	A_84_P14131	1.011	1.281	1.278	0.267	-0.003	Raffinose synthase family protein
AT5G25240	A_84_P78199	1.171	1.291	1.978	0.807	0.687	stress induced protein
AT5G41080	A_84_P56650	1.211	1.760	1.085	-0.127	-0.675	GLYCEROPHOSPHODIESTER PHOSPHODIESTERASE 2 (GDPD2), a member of the PLC-like GDPD family
AT5G41080	A_84_P790164	1.383	1.884	2.277	0.894	0.393	GLYCEROPHOSPHODIESTER PHOSPHODIESTERASE 2 (GDPD2), a member of the PLC-like GDPD family
AT5G45340	A_84_P12143	1.654	3.480	3.274	1.620	-0.206	cytochrome P450, family 707, subfamily A, polypeptide 3
AT5G47600	A_84_P575328	1.099	4.607	5.280	4.182	0.673	HSP20-like chaperones superfamily protein

AT5G50790	A_84_P15944	1.684	1.045	1.572	-0.112	0.527	Nodulin MtN3 family protein
AT5G67300	A_84_P13173	1.342	1.628	1.718	0.376	0.090	myb domain protein r1
AT5G67300	A_84_P807259	1.444	1.680	1.393	-0.051	-0.287	myb domain protein r1

[a]Highted in yellow are loci showed further enhanced expression in double mutant in comparison with single mutants.
