# Supplementary material for: The Arabidopsis Deubiquitylase OTU5 Suppresses Flowering by Histone Modification-Mediated Activation of the Major Flowering Repressors FLC, MAF4, and MAF5
Source: Int J Mol Sci. 2023 Mar 24;24(7):6176. doi: 10.3390/ijms24076176 (PMC10093928; doi:10.3390/ijms24076176)
Supplement: Supplementary file 1 [file ijms-24-06176-s001.zip › TableS08.pdf]

Table S8. Comparison of log_2 fold change of 30 downregulated loci that are overlapped between *otu5*, *arp6*, and *otu5 arp6* mutants[a].

Locus	probe ID	*otu5*	*arp6*	*db*	*db - otu5*	*db - arp6*	description
AT1G10585	A_84_P237173	-1.838	-1.473	-1.501	0.337	-0.028	basic helix-loop-helix (bHLH) DNA-binding superfamily protein
AT1G13470	A_84_P562126	-2.236	-2.217	-2.792	-0.556	-0.575	hypothetical protein (DUF1262)
AT1G13470	A_84_P788748	-2.777	-2.316	-3.330	-0.553	-1.014	hypothetical protein (DUF1262)
AT1G17840	A_84_P816034	-2.034	-1.915	-2.177	-0.143	-0.263	white-brown complex-like protein
AT1G32350	A_84_P21945	-1.868	-1.303	-2.013	-0.145	-0.710	alternative oxidase 1D
AT1G33730	A_84_P11346	-1.140	-1.098	-1.065	0.075	0.033	cytochrome P450, family 76, subfamily C, polypeptide 5
AT1G33960	A_84_P23754	-1.946	-1.606	-1.727	0.219	-0.121	P-loop containing nucleoside triphosphate hydrolases superfamily protein
AT1G49570	A_84_P23433	-1.509	-1.990	-3.017	-1.508	-1.027	Peroxidase superfamily protein
AT1G54230	A_84_P18149	-1.014	-1.324	-1.316	-0.302	0.008	Winged helix-turn-helix transcription repressor DNA-binding protein
AT1G66100	A_84_P15283	-1.147	-1.533	-4.017	-2.871	-2.484	A PR protein belongs to the plant thionin (PR-13) family with multiple members
AT1G66570	A_84_P151538	-1.435	-1.165	-1.503	-0.069	-0.338	sucrose-proton symporter 7
AT1G69530	A_84_P16197	-1.116	-1.636	-1.864	-0.748	-0.228	Expansin A1, a member of alpha-expansin gene family
AT1G69530	A_84_P815585	-1.110	-1.665	-1.926	-0.815	-0.260	Expansin A1, a member of alpha-expansin gene family
AT2G07030	A_84_P15356	-1.445	-1.417	-1.561	-0.116	-0.144	A mutator-like transposase family member
AT2G16005	A_84_P513580	-1.512	-1.276	-1.741	-0.229	-0.465	INTERACTOR OF SYNAPTOTAGMIN1/ROSY1, a MD-2-related lipid recognition domain-containing protein
AT2G19796 AT2G19802	A_84_P857107	-1.124	-1.047	-1.066	0.057	-0.020	transmembrane protein
AT2G29460	A_84_P14441	-1.937	-1.458	-2.003	-0.067	-0.545	glutathione S-transferase tau 4
AT2G44240	A_84_P81249	-2.350	-2.574	-2.894	-0.544	-0.320	NEP-interacting protein (DUF239)
AT3G04717	A_84_P760629	-1.128	-1.167	-1.475	-0.348	-0.308	pseudogene of pathogenesis-related 4
AT3G27884	A_84_P761753	-1.259	-1.067	-1.601	- 0.342	- 0.534	SHORT OPEN READING FRAME 3 (SORF3), identified as a translated small open reading frame by ribosome profiling
AT3G49340	A_84_P20322	-1.904	-1.184	-1.877	0.027	- 0.693	Cysteine proteinases superfamily protein
AT3G49540	A_84_P566998	-1.844	-1.049	-1.239	0.605	- 0.190	hypothetical protein

AT3G49620	A_84_P20323	-2.336	-2.426	-3.800	- 1.464	- 1.374	2-oxoglutarate (2OG) and Fe(II)-dependent oxygenase superfamily protein
AT5G10140	A_84_P10170	-2.376	-1.744	-3.343	- 0.968	- 1.600	FLOWERING LOCUS C (FLC)
AT5G10140	A_84_P784210	-2.282	-1.776	-2.941	- 0.659	- 1.165	FLOWERING LOCUS C (FLC)
AT5G10140	A_84_P784401	-2.381	-1.780	-3.527	- 1.146	- 1.747	FLOWERING LOCUS C (FLC)
AT5G24200	A_84_P585099	-1.793	-1.611	-1.775	0.018	- 0.164	alpha/beta-Hydrolases superfamily protein
AT5G25640	A_84_P558384	-1.027	-1.398	-1.610	- 0.582	- 0.212	Rhomboid-related intramembrane serine protease family protein
AT5G26270	A_84_P500750	-1.591	-1.879	-1.657	- 0.066	0.222	transmembrane protein
AT5G35607	A_84_P310863	-1.320	-1.205	-1.613	- 0.293	- 0.408	A mutator-like transposase family member
AT5G42280	A_84_P23480	-4.194	-3.982	-4.412	- 0.218	- 0.429	Cysteine/Histidine-rich C1 domain family protein
AT5G47990	A_84_P18772	-1.118	-1.151	-1.121	- 0.003	0.030	cytochrome P450, family 705, subfamily A, polypeptide 5
AT5G47990	A_84_P824880	-1.078	-1.084	-1.480	- 0.402	- 0.396	cytochrome P450, family 705, subfamily A, polypeptide 5
AT5G48000	A_84_P19718	-1.020	-1.167	-1.177	- 0.158	- 0.011	cytochrome P450, family 708, subfamily A, polypeptide 2

[a]Highted in yellow are loci showed further reduced expression in double mutant in comparison with single mutants.
